# Colchicine in atrial fibrillation: mechanisms, highlights, and current evidence

**DOI:** 10.3389/fphar.2026.1857470

**Published:** 2026-07-17

**Authors:** Xiao-Xue Guo, Chu Zhang, Hai-Juan Wang, Zhao-Fen Wang, Peng Chang

**Affiliations:** 1 Second Clinical Medical College, Lanzhou University, Lanzhou, Gansu, China; 2 Department of Cardiology, Lanzhou University Second Hospital, Lanzhou, China

**Keywords:** atrial fibrillation, colchicine, inflammation, mechanisms, surgery

## Abstract

Currently, the global incidence of atrial fibrillation (AF) is rising, and its associated complications, such as stroke, heart failure, and thromboembolism, severely impair patients’ quality of life and increase mortality and healthcare burdens. With the in-depth investigation into the mechanisms underlying the initiation and maintenance of AF, apart from classic mechanisms such as electrical remodeling, structural remodeling and autonomic nervous system dysfunction, the role of inflammatory response in the pathogenesis of AF has attracted increasing attention. Colchicine has been used as an anti-inflammatory agent for the treatment of gout and familial Mediterranean fever for many years, and it has shown favorable safety and tolerability. With continuous research, it has also demonstrated great potential in cardiovascular diseases and has been applied to the treatment and prevention of pericarditis and coronary heart disease. This article aims to summarize the mechanisms underlying colchicine’s prevention of AF, discuss dosage applications for AF, outline the accumulated evidence for colchicine in preventing AF across different surgical types and disease categories, and provide an outlook on future research directions.

## 1 Introduction

Atrial fibrillation (AF) is one of the most common types of arrhythmias driven by intricate pathophysiological mechanisms. Over the past 3 decades, the global burden of atrial fibrillation and atrial flutter (AF/AFL) has been increasing. In 2021, approximately 52.6 million people worldwide were living with AF/AFL. The prevalence has risen more rapidly among women, and it is projected that the age-standardized prevalence rate (ASPR) and age-standardized incidence rate (ASIR) in females will continue to increase from 2022 to 2046 (Tan et al., 2025). Thus, there is an urgent need to improve the management of the onset and progression of AF. The main treatment measures for AF are currently controlling the ventricular rate, restoring and maintaining sinus rhythm, and preventing thromboembolism; however, these strategies have limitations such as poor efficacy and multiple adverse reactions. AF upstream therapy, which was first introduced in the management of AF in 2010, refers to early intervention targeting the etiology and mechanisms underlying AF initiation, thereby preventing or delaying the development and progression of the disease at its source (Camm et al., 2010). With the exploration of the mechanisms of AF, inflammation has become a hot topic. The unique anti-inflammatory effect of colchicine and its advantage of low cost make it a potentially useful oral cardiovascular treatment for AF. Colchicine has a long history in medicine, having been used for gout and Familial Mediterranean Fever for many years. In recent years, colchicine has also been used in the treatment and prevention of pericarditis and chronic coronary syndromes (CCS) (Adler et al., 2015; Vrints et al., 2024). Colchicine is mainly absorbed in the jejunum and ileum, with a bioavailability of 25%–50% when taken orally (Slobodnick et al., 2018; Ben-Chetrit and Levy, 1998). Its most common adverse reactions are gastrointestinal, including abdominal pain, diarrhea, nausea, and vomiting (Stewart et al., 2020). Colchicine exerts a dose-dependent effect, and most side effects are reversible upon dose reduction or discontinuation of treatment (Wołowiec et al., 2025). Colchicine is primarily excreted via the liver, though renal excretion also occurs. In the liver, it undergoes demethylation mediated by P450 3A4 (CYP3A4) and is then excreted through the biliary tract and urinary tract (Tateishi et al., 1997; Leighton et al., 1991). P-glycoprotein (P-gp), an ATPase-dependent efflux pump, exerts biological effects by reducing cellular uptake and facilitating the excretion of colchicine (Declèves et al., 2006). Therefore, concomitant use with drugs that inhibit CYP3A4 or P-gp, such as ketoconazole, cimetidine, imatinib, verapamil, and amiodarone, may enhance the toxicity of colchicine.

## 2 The pathogenic mechanism of AF

The core feature of AF is the disorder of atrial electrical activity, which impairs the regular contraction of the atria. Instead, there is rapid and disorganized electrical fibrillation, which in turn leads to an absolutely irregular heart rhythm. The pathogenic mechanism of AF is complex and multifactorial, involving the development of atrial cardiomyopathy (AtCM). The presence of AtCM may provide a substrate for the development of AF, while AF in turn can significantly accelerate the progression of AtCM (Goette et al., 2024).

### 2.1 Atrial remodeling

#### 2.1.1 Structural remodeling

Atrial fibrosis is recognized as a key factor in AF and its associated complications, and its relationship with AF has been thoroughly described in relevant review articles (Karakasis et al., 2024; Boyle et al., 2021; Horrach et al., 2025). The mechanisms of atrial fibrosis are diverse, including pressure overload, neurohumoral activation, inflammation, oxidative stress, and possibly even atrial fibrillation itself, in which cardiac fibroblasts play a pivotal role (Karakasis et al., 2024; Heijman et al., 2021; Pang et al., 2025). Non-myocytes account for approximately 65%–70% of total cardiac cells. Among these cell populations, endothelial cells make up more than 60%, and cardiac fibroblasts constitute 15%, the latter representing the main cellular source of extracellular matrix (ECM) proteins (Nag, 1980; Pinto et al., 2016). Cardiac fibroblasts account for approximately 15% of non-cardiac cells and serve as the primary source of extracellular matrix (ECM) proteins (Yue et al., 2011). In pathological states, cardiac fibroblasts trigger a different proportion of collagen subtypes compared to healthy hearts (Xu et al., 2004). A comparison of right atrial appendage tissues from patients undergoing cardiac surgery revealed that the ratio of type III collagen (Col III) to type I collagen (Col I) was significantly lower in patients who developed postoperative atrial fibrillation (POAF) than in those who maintained postoperative sinus rhythm (Grammer et al., 2005). In histopathological classification, myocardial fibrosis is categorized into two subtypes: reparative fibrosis and reactive fibrosis (De Sensi et al., 2021). Reparative fibrosis is triggered by the necrosis or apoptosis of cardiomyocytes, which induces the proliferation of fibroblasts and subsequent collagen secretion to fill defective tissue areas and form scar tissue. This pathological process directly impairs the electrical interconnections among cardiomyocytes, thereby resulting in cardiac conduction block (Nattel, 2017). Reactive fibrosis arises from pressure overload or cardiac inflammation, encompassing perivascular fibrosis surrounding muscle bundles and endomysial fibrosis between cardiomyocytes (Horrach et al., 2025; de Boer et al., 2019). Compared to reparative fibrosis, endomysial fibrosis plays a crucial role in atrial conduction (Maesen et al., 2022). It disrupts transverse muscle connections and facilitates the propagation of irregular electrical waves, thereby establishing re-entrant circuits that sustain atrial fibrillation (Horrach et al., 2025).

#### 2.1.2 Electrical remodeling

The maintenance of AF is mainly dependent on focal ectopic activity and re-entry mechanisms. Atrial ectopic electrical activity arises from multiple mechanisms, among which triggered activity induced by delayed afterdepolarizations (DADs) and early afterdepolarizations (EADs) represents the most important one (Lip et al., 2016). L-type calcium current (I_CaL_) drives the process of ‘Ca^2+^ induced Ca^2+^ release’ mediated by the ryanodine receptor 2 (RyR2), which constitutes the core mechanism of myocardial contraction. Enhanced Ca^2+^ sensitivity of RyR2 or sarcoplasmic reticulum (SR) Ca^2+^ overload can trigger abnormal Ca^2+^ release from the SR during diastole, thereby leading to DADs (Nattel and Dobrev, 2012). The recovery of I_CaL_ from inactivation, increased activity of the Na^+^/Ca^2+^ exchanger (NCX), and enhanced Ca^2+^/calmodulin-dependent protein kinase type II (CaMKII) activity all contribute to the occurrence of EADs (Nattel and Dobrev, 2012). Currently, two main conceptual models are recognized to underlie re-entry: the ‘leading circle’ and the ‘spiral wave’. In the ‘leading circle’ model, the stability of AF is determined by the number of re-entrant circuits that can be sustained within the atria. When the wavelength (WL = RP × CV, where WL denotes wavelength, RP represents refractory period, and CV indicates conduction velocity) is shortened or atrial dilation occurs, the atria can accommodate a greater number of re-entrant circuits, thereby facilitating the self-perpetuation of AF. Similarly, in the ‘spiral wave’ concept, a reduction in RP promotes the initiation and maintenance of spiral wave re-entry (Lip et al., 2016). Reduction in I_CaL_ and increased outward K^+^ current directly shorten the action potential duration (APD). This change may reduce the atrial RP, which in turn significantly promotes the formation of re-entry (Nattel and Dobrev, 2012). Recently, the emerging role of small conductance Ca^2+^-dependent K^+^ channels (SK channels) in AF has gained increasing attention. SK channels are expressed in cardiomyocytes of the atria, ventricles, and cardiac conduction system, with particularly prominent expression in atrial and pulmonary vein cardiomyocytes (PVCs) (Liu et al., 2023). Although the precise mechanism underlying the function of SK channels remains unclear, numerous studies have demonstrated their complex regulatory effects in the pathogenesis of AF (Heijman et al., 2023).

### 2.2 Inflammation

Inflammation serves as a defensive mechanism for the body, activated in response to tissue injury or infection; however, it can cause varying degrees of damage to the body when the inflammatory response becomes unbalanced. In an immunohistochemical analysis of intraoperative atrial biopsy specimens from 46 subjects undergoing coronary bypass or valve surgery, among which 19 were diagnosed with AF and 27 with sinus rhythm, inflammatory cell infiltration was found to be independently associated with AF (Smorodinova et al., 2017). A large prospective cohort study involving over 400,000 individuals with a follow-up period exceeding 12 years analyzed the association between systemic inflammatory markers, including C-reactive protein (CRP), neutrophils, monocytes, and lymphocytes, and arrhythmic outcomes such as AF, demonstrating a significant association between systemic inflammation levels and the risk of arrhythmias (Yang et al., 2023). The NACHT, leucine-rich repeat (LRR), and pyrin domain (PYD)-containing protein 3 (NLRP3) inflammasome is a multiprotein complex that is expressed in both immune and non-immune cell types, and it coordinates the innate immune responses to infectious and sterile stimuli (Karakasis et al., 2025). Excessive activation of the NLRP3 inflammasome is associated with chronic low-grade inflammation, and a growing body of evidence has indicated that NLRP3 serves as a crucial driver in the initiation and progression of AF (Dobrev et al., 2023). Elevated extracellular ATP, intracellular Ca^2+^ mobilization, oxidative stress, lysosomal rupture, autophagy inhibition, and mitochondrial dysfunction are considered relevant factors involved in the activation of the NLRP3 inflammasome (Toldo et al., 2022). Activation of NLRP3 promotes the maturation of pro-caspase-1, which in turn induces the secretion of the proinflammatory cytokines interleukin-1β (IL-1β) and interleukin-18 (IL-18), while elevated levels of IL-18 could further drive the transition from paroxysmal AF to persistent AF (Karakasis et al., 2025; Luan et al., 2010). The NLRP3 inflammasome also exerts a certain effect on atrial fibrosis. In a rat model of fecal microbiota transplantation (FMT), elderly FMT rats treated with the inflammasome inhibitor MCC950 exhibited reduced atrial collagen deposition, a lower collagen volume fraction, and decreased expression of fibrosis-related genes (Zhang et al., 2022). In a mouse model with cardiomyocyte-specific knock-in of constitutively active NLRP3 (CM-KI), NLRP3 activation promotes SR Ca^2+^ release, upregulates total protein expression of RyR2 in atrial tissue, and shortens the atrial effective refractory period (AERP), thereby facilitating atrial electrical remodeling (Yao et al., 2018). EAT, a fat deposit located between the myocardium and the epicardium, acts as an active paracrine and immunoregulatory organ that participates in the pathophysiological processes of AF, and it can also secrete a series of proinflammatory adipokines to form a local proinflammatory microenvironment (Mazurek et al., 2003; Iacobellis, 2022). Burg et al. reported that in a systolic HF rat postmyocardial infarction (post-MI) model, treatment with BA6b9, an allosteric inhibitor of the SK4 channels, resulted in reduced left atrial collagen deposition, α-smooth muscle actin (α-SMA) expression, upregulation of NLRP3, as well as decreased AF induction and AF duration (Burg et al., 2024).

## 3 The relevant mechanism of colchicine

**FIGURE 1:**
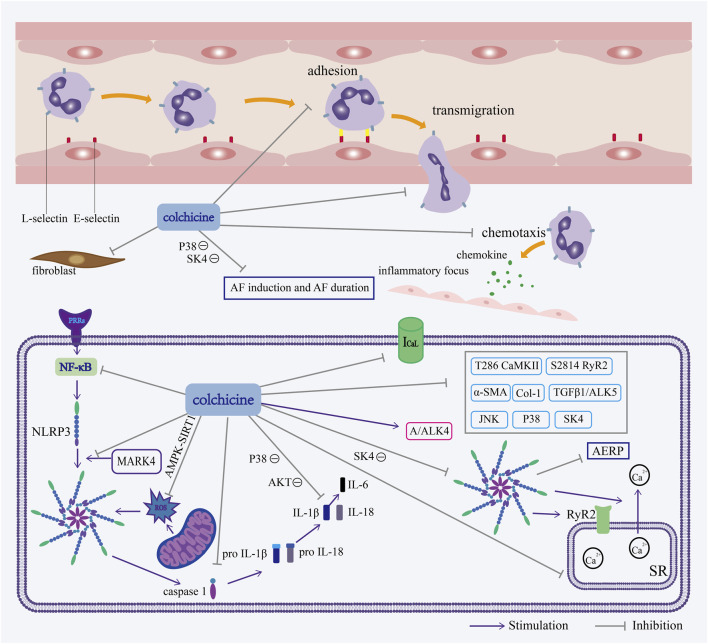
Mechanisms of action of colchicine. (1) Colchicine regulates the quantity and distribution of selectins on leukocytes and endothelial cells, thereby modulating leukocyte adhesion. It alters leukocyte deformability by interfering with microtubules. Colchicine can also inhibit leukocyte chemotaxis (2). Colchicine suppresses the assembly and activation of the NLRP3 inflammasome, as well as the subsequent release of downstream pro-inflammatory cytokines (3). By inhibiting the NLRP3 inflammasome, colchicine attenuates NLRP3-mediated atrial remodeling effects. Additionally, colchicine downregulates the expression of genes associated with atrial remodeling, thereby improving atrial remodeling.

Colchicine exerts its effects primarily through its regulatory capacity on microtubules (Figure 1). As a key structural component of the cellular cytoskeleton, microtubules play a pivotal role in maintaining cellular morphology, mediating cellular movement and division, as well as facilitating intracellular material transport. Colchicine binds irreversibly to soluble tubulin heterodimers to form a colchicine-tubulin complex (TC), thereby inhibiting microtubule assembly (Bergen and Borisy, 1983; Hastie, 1991).

### 3.1 Inhibit inflammatory cells

The primary pharmacological target of colchicine is leukocytes, with the peak plasma concentration of colchicine typically occurring 48 h after oral administration (Chappey et al., 1993). Colchicine exerts its anti-inflammatory effects by inhibiting leukocyte responses during inflammation. At lower doses, colchicine modulates the distribution of E-selectin molecules on the surface of endothelial cells, while higher doses further downregulate the expression of L-selectin on neutrophils, thereby impairing neutrophil adhesion (Cronstein et al., 1995). Additionally, colchicine reduces the deformability of neutrophils by promoting microtubule depolymerization and enhancing the polymerization of actin, which in turn restricts neutrophil transmigration during inflammation (Paschke et al., 2013). In mice with early atherosclerosis (Apoe^−/−^ mice fed a high-cholesterol diet for 8 consecutive weeks), colchicine was found to inhibit leukocyte chemotaxis by suppressing the leukocyte recruitment phenotype, and to reduce the numbers of neutrophils, inflammatory monocytes, and macrophages within the atherosclerotic aorta (Meyer-Lindemann et al., 2022). Similarly, in a rat model of heart failure, colchicine decreased the infiltration of macrophages and neutrophils in myocardial tissue (Shen et al., 2022).

### 3.2 Inhibit the NLRP3 inflammasome

The core mechanism by which colchicine exerts its effects on the NLRP3 inflammasome is the inhibition of NLRP3 inflammasome activation and downstream inflammatory responses. Specifically, colchicine exerts this effect by interfering with microtubule-dependent assembly of the NLRP3 inflammasome, suppressing the activation of the NLRP3 inflammasome, and reducing the release and secretion of downstream pro-inflammatory cytokines (Li et al., 2017; Misawa et al., 2013; Yang et al., 2020; Otani et al., 2016). As the assembly of the NLRP3 inflammasome is microtubule-dependent, colchicine can impair the assembly of the NLRP3 complex by decreasing the interaction between microtubule affinity-regulating kinase 4 (MARK4) and NLRP3 (Li et al., 2017; Misawa et al., 2013). The activation of the NLRP3 inflammasome consists of two distinct phases: priming and triggering. During the priming phase, the binding of pathogen-associated molecular patterns (PAMPs) and damage-associated molecular patterns (DAMPs) to Toll-like receptors (TLRs) stimulates the NF-κB signaling pathway, leading to the transcriptional upregulation of NLRP3 (Karakasis et al., 2025). Multiple studies have reported the inhibitory effect of colchicine on NF-κB (Cimmino et al., 2021; Ben-Dav et al., 2018). In human umbilical vein endothelial cells, colchicine was found to upregulate the expression and activity of various antioxidant enzymes and reduce ROS production by activating the AMPK-SIRT1 pathway, thereby suppressing NLRP3 inflammasome activation (Yang et al., 2020). Additionally, in research on non-steroidal anti-inflammatory drug (NSAID)-induced small intestinal injury, colchicine was shown to inhibit the protein expression of caspase 1 and mature IL-1β without altering NLRP3 mRNA levels, which are the downstream products of NLRP3 (Otani et al., 2016). Furthermore, colchicine could suppress IL-6 secretion induced by IL-1β through inhibiting the activation of P38, JNK, AKT, and NF-κB p65 signaling pathways (Wu et al., 2020).

### 3.3 Attenuate atrial remodeling

Colchicine has been demonstrated to exert an inhibitory effect on renal fibrosis by disrupting microtubules and suppressing hepatic fibrosis through inhibiting the activation of hepatic stellate cells (Guan et al., 2013; Shu et al., 2009). Current research has verified the efficacy of colchicine in alleviating myocardial inflammatory responses and the progression of myocardial fibrosis from the aspects of cardiac function, histopathology, and ultrastructure (Chen et al., 2025). In an experimental study on the inhibitory effect of colchicine on AF using a rat model of sterile pericarditis (SP), it was found that colchicine could reduce atrial fibrosis and the expression of fibrosis-related genes, such as α-smooth muscle actin (α-SMA, a marker of fibroblast activation) and Col-1 (Wu et al., 2020). Meanwhile, it may alleviate atrial fibrosis by inhibiting the activation of AKT and P38 signaling pathways (Wu et al., 2020). JNK and p38 belong to the family of stress-activated protein kinases. JNK has been also implicated in the pathogenesis of myocardial fibrosis (Wu et al., 2011). However, Wu et al. did not detect any significant effect of colchicine on JNK in rats of SP. In contrast, Levi et al. observed a modulatory effect of colchicine on JNK in post- MI rats with reduced ejection fraction, yet no beneficial impact of colchicine on myocardial fibrosis was demonstrated in that study (Wu et al., 2020; Levi et al., 2026). Yue et al. observed in their study on a rat AF model that colchicine could attenuate myocardial fibrosis via inhibiting the TGFβ1/ALK5 fibrosis pathway and activating the ALK4 fibrosis pathway (Yue et al., 2022). In addition, colchicine can also inhibit the activation, phenotypic transformation, and apoptosis resistance of cardiac fibroblasts, and reduce their capacity for synthesizing inflammatory factors and collagen (Yue et al., 2022).

Colchicine has been shown to attenuate atrial electrical remodeling. In the study conducted on HL-1 cardiomyocytes, colchicine has been demonstrated to reduce intracellular Ca^2+^ transients and SR Ca^2+^ content, thereby mitigating Ca^2+^ overload (Lu et al., 2016). In a mouse model of inflammation induced by lipopolysaccharide (LPS), colchicine was observed to restore ion channels and mitigate the downregulation of Ca^2+^-handling proteins seen in the LPS group, and to reduce the expression level s of phosphorylated CaMKII at the T286 site and phosphorylated RyR2 at the S2814 site (Ying et al., 2023). As mentioned above, colchicine may also potentially suppress electrical remodeling via inhibiting the NLRP3 inflammasome. Colchicine has been shown to exert effects on JNK, p38, and SK4 channels. However, it remains unclear whether colchicine modulates atrial electrical remodeling through its actions on these molecular targets. Studies have demonstrated that JNK phosphorylates CaMKII and promotes abnormal diastolic SR Ca^2+^ leakage. Meanwhile, JNK reduces connexin 43 (Cx43) in gap junctions, leading to slowed conduction and increased AF susceptibility (Yan et al., 2018a; Yan et al., 2018b). In rat models of AF, treatment with a P38 MAPK inhibitor has been observed to prolong AERP, shorten AF duration, and reduce the inducibility of AF (Wang et al., 2025). Inhibitor of the SK4 channels has been shown to prolong AERP, thereby reducing re-entry (Burg et al., 2024). Nevertheless, the study by Levi et al. did not demonstrate a prolonging effect of colchicine on AERP (Levi et al., 2026).

## 4 Application dose of colchicine for preventing AF

Colchicine’s effective pharmacological site is leukocytes, and as extracellular colchicine levels continue to rise, the uptake of colchicine by leukocytes tends to plateau (Chappey et al., 1993). The AGREE study focusing on acute gout flare recurrence demonstrated that the peak efficacy was comparable between the low-dose colchicine regimen (total 1.8 mg within 1 h) and the high-dose regimen (total 4.8 mg within 6 h), while the former experienced fewer adverse reactions than the latter (Terkeltaub et al., 2010). It indicates that increasing the dosage of colchicine does not lead to enhanced therapeutic efficacy, suggesting that low-dose colchicine may possess more favorable clinical applicability. In a mouse model of LPS-induced inflammation, colchicine treatment exhibited a U-shaped dose-response relationship, with a significant improvement in survival observed only at doses ranging from 0.10 to 0.40 mg/kg (Ying et al., 2023). Similarly, in a rat model of sterile pericarditis, among four tested dosages (0.01, 0.1, 0.5, and 1 mg kg^-1^·d^-1^), 0.5 mg kg^-1^·d^-1^ was identified as the optimal dose for suppressing AF. A higher dosage (1 mg kg^-1^·d^-1^) resulted in a marked reduction in heart rate (393.8 ± 14.1 bpm vs. 442.0 ± 11.4 bpm in the saline group, P < 0.01) without further enhancement of the anti-AF effect. In contrast, lower dosages (0.01 and 0.1 mg kg^-1^·d^-1^) led to attenuated or abolished anti-AF activity (Wu et al., 2020). Therefore, lower doses of colchicine are not necessarily better. The optimal dosage for AF patients requires further exploration. The therapeutic plasma concentration range of colchicine is 0.5–3 μg/L, and toxic reactions typically occur when the concentration exceeds 3 μg/L (Stamp et al., 2024). When colchicine is administered at a daily dose of 0.5–0.6 mg, serum levels remain below the upper safety threshold of 3.0 μg/L in the absence of advanced renal or hepatic disease (Robinson et al., 2022). According to the 2024 ESC Guidelines for the management of CCS, low-dose colchicine 0.5 mg once daily should be considered in CCS patients with atherosclerotic coronary artery disease (CAD) to reduce the risk of myocardial infarction, stroke, and the need for revascularization, which is classified as Class IIa, Level A (Vrints et al., 2024). Mohammed Al-Sadawi et al. reported that in patients undergoing catheter ablation (CA), administration of low-dose colchicine (0.3–0.6 mg once daily) for 1 month was associated with a significant reduction in AF recurrence, along with higher patient adherence (Al-Sadawi et al., 2025). In current studies investigating the efficacy of colchicine in the management of AF, the commonly adopted dosage regimen is 0.5 or 0.6 mg administered twice daily. However, this dosing strategy is associated with a relatively high incidence of adverse reactions, which in turn leads to a high rate of treatment discontinuation. Tabbalat et al. investigated the effect of colchicine on the incidence of AF in patients undergoing open heart surgery in 2016 and 2020, respectively. In the 2016 study, the incidence of diarrhea was 24.6% in the colchicine group (0.5 mg twice daily) versus 5.5% in the placebo group, while in the 2020 study, the incidence of diarrhea was 2% in the colchicine group (0.5 mg once daily) versus 3% in the placebo group (Tabbalat et al., 2016; Tabbalat et al., 2020). Tian et al. conducted an analysis to evaluate the efficacy and safety of colchicine for the prevention of AF, which included a total of 17 randomized controlled trials (RCTs) involving 16,238 patients, demonstrating that colchicine exhibited favorable efficacy in reducing the incidence of AF, and low-dose (0.5 mg once daily) and long-term use of colchicine was associated with a lower risk of gastrointestinal adverse events (Tian et al., 2024). Therefore, low-dose colchicine (0.5 mg once daily) may be more suitable for the prevention and treatment of AF. Further studies are still required to explore its optimal dosage and combination therapy.

## 5 The role of colchicine in postoperative atrial fibrillation

**FIGURE 2:**
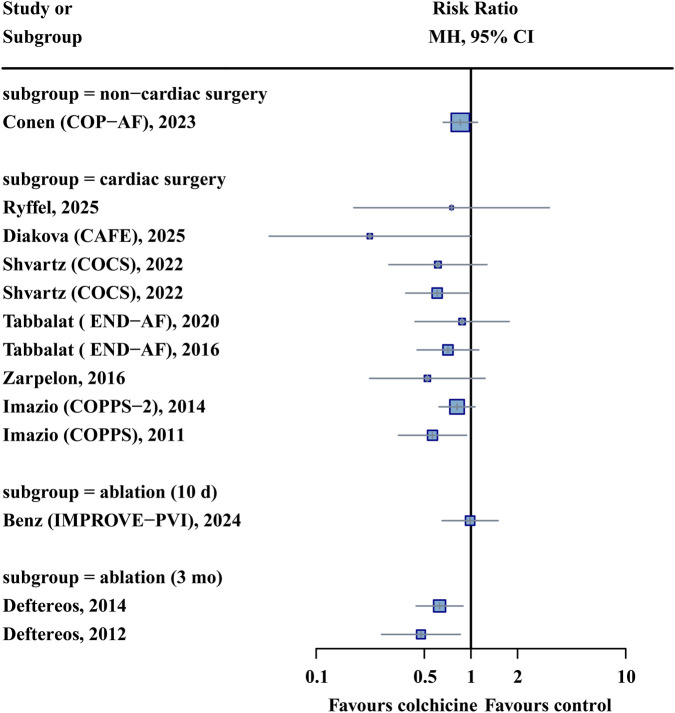
Forest plot of the effectiveness of colchicine in different types of surgeries. Colchicine seems to offer substantial benefits in reducing POAF. It exhibits a protective effect in patients undergoing cardiac surgery and at 3 months after catheter ablation, whereas no significant benefit is detected in the non-cardiac surgery and in the early post-ablation period subgroup.

POAF is the most prevalent form of secondary AF, which is associated with prolonged hospital stays, elevated hospitalization costs, and increased risks of adverse cardiovascular events, stroke, and mortality (Caldonazo et al., 2023; Lin et al., 2019). Numerous studies have investigated the preventive efficacy of colchicine against POAF (Table 1; Figure 2).

**TABLE 1:** Summary of studies investigating colchicine as adjunct in POAF.

Study	Type	Surgery	No. of patients	Intervention	Conclusion (efficiency of reducing AF)
Conen et al. (2023)	RCT	non-cardiac surgery	3,209	Colchicine 0.5 mg BID for 10 days	NO
Ryffel et al. (2025)	RCT	TAVR	120	N/A	NO
Diakova et al. (2025)	RCT	CABG	140	Colchicine 0.5 mg 4 h preoperatively and then 0.5 mg BID until 10 days postoperatively	YES
Shvartz et al. (2022a)	RCT	CABG/AVR	101	Colchicine 1 mg 24 h preoperatively until 5 days postoperatively	NO
Shvartz et al. (2022b)	RCT	CABG/AVR	240	Colchicine 1 mg 24 h preoperatively until 5 days postoperatively	YES
Tabbalat et al. (2020)	RCT	Cardiac surgery	152	Colchicine 1 mg 12–24 h preoperatively and then 0.5 mg QD until hospital discharge	NO
Tabbalat et al. (2016)	RCT	Cardiac surgery	360	Colchicine 2 mg 12–24 h preoperatively and 1 mg 4 h before or immediately after surgery and then 0.5 mg BID until hospital discharge	NO
Zarpelon et al. (2016)	RCT	myocardial revascularization surgery	140	Colchicine 1 mg BID 24 h preoperatively and then 0.5 mg BID until hospital discharge	NO
Imazio et al. (2014)	RCT	Cardiac surgery	360	Colchicine 0.5 mg BID in patients ≥70 kg or 0.5 mg QD in patients <70 kg for 1 month	NO
Imazio et al. (2011)	RCT	Cardiac surgery	336	Colchicine 1 mg BID starting on postoperative day 3 followed by a maintenance dose of 0.5 mg BID in patients ≥70 kg, halved doses for patients <70 kg or intolerant to the highest dose for 1 month	YES
Be et al. (2024)	RCT	CA	199	Colchicine 0.6 mg BID for 10 days	NO
De et al. (2014)	RCT	CA	206	Colchicine 0.5 mg BID for 3 months	YES
Deftereos et al. (2012)	RCT	CA	161	Colchicine 0.5 mg BID for 3 months	YES
Romero et al. (2025)	Prospective	CA	129	NA	YES
Mohanty et al. (2023)	Prospective	CA	1,075	Colchicine 0.3 mg BID for 1 month	YES
Al-Sadawi et al. (2025)	Retrospective	CA	550	Colchicine 0.3–0.6 mg QD for 30 days	YES
Campbell et al. (2023)	Retrospective	CA	180	Colchicine 0.6 mg QD for 30 days	NO

RCT, randomized controlled trial; TAVR, transcatheter aortic valve replacement; CABG, coronary artery bypass grafting; AVR, aortic valve replacement; CA, catheter ablation; NA, not available.

### 5.1 For cardiac surgery

POAF occurs in approximately 30% of patients following cardiac surgery, with coronary artery bypass grafting (CABG) accounting for about 20% of cases, valve surgery for 40%–50%, aortic surgery for approximately 30%, and heart transplantation for about 4% (Gaudino et al., 2023). In a comparative study using a rat model of sterile pericarditis to investigate the efficacy of amiodarone, colchicine, and extracellular vesicles (EVs) derived from human cardiac cells in preventing POAF, colchicine demonstrated a positive trend toward reducing the incidence of POAF, although this trend was not statistically significant (Parent et al., 2024). However, this study mainly focused on EVs rather than colchicine, which may lead to some bias in its results. In 2011, a subgroup analysis of the COPPS randomized controlled trial focusing on AF demonstrated that colchicine reduced the incidence of POAF in patients undergoing cardiac surgery (12.0% versus 22.0%) (Imazio et al., 2011). Nevertheless, subsequently in 2014, the COPPS-2 trial showed that perioperative colchicine did not significantly decrease the occurrence of POAF (33.9% versus 41.7%), although a trend toward a lower incidence was observed (Imazio et al., 2014). This discrepancy may be attributed to the higher rate of early discontinuation of colchicine in the COPPS-2 trial. A meta-analysis incorporating COPPS and COPPS-2 trials demonstrated that perioperative colchicine administration may reduce the incidence of POAF (RR: 0.65) (Lee et al., 2016). It should be noted that these two RCTs enrolled patients undergoing various types of cardiac surgery, resulting in substantial heterogeneity; therefore, their findings should be interpreted with caution. Regarding CABG, Diakova et al. administered colchicine at a dose of 0.5 mg preoperatively and 0.5 mg twice daily postoperatively in patients with CAD, combined with an original intraoperative pericardial fenestration technique. Their results demonstrated a favorable preventive effect, with an incidence of 2.9% in the colchicine group versus 12.9% in the control group (P = 0.03). Since the study incorporated an original surgical intervention, the independent therapeutic effect of colchicine could not be precisely determined (Diakova et al., 2025). In a double-blind, randomized, placebo-controlled trial, colchicine was found to reduce the composite endpoint events, which included new-onset AF and atrioventricular block requiring permanent cardiac pacemaker implantation, following transcatheter aortic valve replacement (TAVR), but no significant reduction in new-onset AF alone was observed (Ryffel et al., 2025). Currently, a large-scale, randomized, double-blind clinical trial involving 8 centers is underway. In this study, low-dose colchicine is administered at 0.5 mg daily for 3 days preoperatively and 0.5 mg every other day for 10 days postoperatively, aiming to investigate the effect of colchicine on the incidence of new-onset AF within 10 days after surgery (Li et al., 2024).

### 5.2 For atrial fibrillation ablation

Increased excitability of cardiomyocytes located near the pulmonary vein ostia (PVs) represents the most common trigger for AF (Sanders et al., 2005). Achieving pulmonary vein isolation (PVI) serves as the fundamental strategy for all CA procedures in patients with AF. In a study focusing on isolated atrial fibrillation, patients scheduled for ablation underwent interatrial septum (IAS) biopsy under transesophageal echocardiography guidance before the procedure. Results indicated that the presence of inflammatory and fibrotic changes in myocardial tissue may contribute to an increased risk of early and late arrhythmia recurrence in patients undergoing ablation (Batalov et al., 2023). Recently, another study investigating the systemic immune-inflammation index (SII) reported that an SII level ≥457.41 × 10^9^/L was associated with a significantly higher rate of AF recurrence, supporting the critical role of inflammation in post-ablation AF recurrence (Zhang et al., 2025). Two RCTs on paroxysmal AF, published in 2012 and 2014 respectively, demonstrated that oral administration of colchicine at a dose of 0.5 mg twice daily within 3 months postoperatively reduces AF recurrence following CA (De et al., 2014; Deftereos et al., 2012). However, the study conducted in 2024 reported conflicting results (Be et al., 2024). This discrepancy may stem from the latter study permitting the use of antiarrhythmic drugs, which could have bridged the gap between the two groups, and having a shorter duration of drug administration (10 days). A recent retrospective cohort study was conducted using the TriNetX Research database, enrolling patients between 2015 and 2024. It investigated the effect of colchicine on AF recurrence following CA, based on colchicine administration within the first month after ablation, which showed a reduced likelihood of requiring cardioversion during the 1-year follow-up period after CA (Kanaan et al., 2025).

### 5.3 For non-cardiac surgery

POAF occurs in approximately 15% of patients following non-cardiac surgery (Gaudino et al., 2023). This estimate may underrepresent the true prevalence, as only a small proportion of patients undergo arrhythmia screening following non-cardiac surgery. Increasing age, extensive surgical procedures, male gender, and stage II or higher tumors are recognized as predictive factors for POAF (Onaitis et al., 2010). A large randomized controlled trial (COP-AF) demonstrated that administration of colchicine 0.5 mg twice daily, initiated 4 h before surgery and continued for 10 days postoperatively, did not prevent POAF in patients undergoing non-cardiac surgery (Conen et al., 2023). This finding may be attributed to the fact that in patients undergoing major non-cardiac thoracic surgery, surgery-related factors, autonomic nervous activity, and atrial load play more dominant roles than local cardiac inflammation in the pathogenesis of POAF.

## 6 The role of colchicine in cardiovascular diseases with atrial fibrillation

Common cardiovascular diseases, including AF, myocardial infarction (MI), stroke, and heart failure (HF), share multiple risk factors such as hypertension, dyslipidemia, obesity, inflammation, diabetes mellitus, and smoking, which may underlie the association among different cardiovascular diseases. In the 40-year follow-up of the ULSAM study, the association between HF and AF demonstrated a very high hazard ratio. Similarly, MI was also associated with a significantly elevated hazard ratio for AF, although the relative risk was lower than that for HF. This association was independent of the sequence of disease onset (Lind et al., 2021). AF has been demonstrated to be an independent risk factor for stroke and acute myocardial infarction (AMI) (Wolf et al., 1991; Soliman et al., 2014). The mechanisms underlying AF after MI have been comprehensively summarized in previous reviews (Frederiksen et al., 2023). Naeem et al. conducted a meta-analysis of treatment efficacy in post-MI patients, incorporating 14 RCTs involving 14,326 participants. The analysis indicated no beneficial effect of colchicine on AF in post-MI patients (Naeem et al., 2025). Current available evidence does not clearly support that colchicine use in MI patients reduces the incidence of AF (Naeem et al., 2025; Nazmy et al., 2025). HF and AF are mutually causal and interact with each other, which is related to their complex pathophysiology. In a rabbit HF model, colchicine can shorten the APD in the left atrial appendage (LAA), reduce triggered activity, and flatten the restitution curve (RC), thereby suppressing the occurrence of arrhythmias and AF (Singhal et al., 2014). Cardioembolic stroke represents a major subtype of ischemic stroke, with cardiac embolism-induced ischemic strokes being more severe than other subtypes. The most common cause is AF, primarily through the interaction of abnormal atrial contractions with other atrial factors (Boyle et al., 2021; Kamel and Healey, 2017; Escudero-Martínez et al., 2023). Currently, anticoagulation remains the mainstay for the prevention of AF-related stroke. However, given the high risks of recurrent stroke and bleeding complications, inflammation-targeted antithrombotic strategies have emerged as novel approaches for stroke prevention (Katsanos et al., 2020). Hagag et al. investigated the efficacy of colchicine in stroke prevention among patients with stroke or at high risk (including CAD, AF, and non-cardioembolic stroke). Although their results did not reach statistical significance, they demonstrated a favorable trend toward stroke reduction (Hagag et al., 2025).

## 7 Conclusion and outlook

Colchicine has shown promising potential in the field of cardiovascular diseases in recent years. Its favorable anti-inflammatory and anti-fibrotic effects provide novel strategies for the prevention and treatment of AF. Existing studies have confirmed that colchicine is associated with beneficial outcomes in both cardiac surgery and AF ablation. However, its efficacy in preventing AF in patients undergoing non-cardiac surgery remains limited. Current research on colchicine for AF prophylaxis in cardiac surgery is characterized by high heterogeneity, warranting further investigations into its therapeutic efficacy across different types of cardiac surgery. Low-dose colchicine appears to exhibit more favorable clinical applicability, yet additional studies are required to determine the optimal initiation time, dosage, duration of treatment, and rational combination regimens of colchicine.
