# Correction: Colchicine in atrial fibrillation: mechanisms, highlights, and current evidence

**DOI:** 10.3389/fphar.2026.1947353

**Published:** 2026-07-31

**Authors:** Frontiers Production Office

**Affiliations:** Frontiers Media SA, Lausanne, Switzerland

**Keywords:** atrial fibrillation, colchicine, inflammation, mechanisms, surgery

Or Levi was erroneously omitted as a junior reviewer in collaboration with Yoram Etzion in the published article. The reviewers’ details have been corrected to read:

Yoram Etzion, Ben-Gurion University of the Negev, Israel.

Or Levi, Ben-Gurion University of the Negev, Israel, in collaboration with reviewer YE.

The original version of this article has been updated.

